# Focused Examination of the Intestinal lamina Propria Yields Greater Molecular Insight into Mechanisms Underlying SIV Induced Immune Dysfunction

**DOI:** 10.1371/journal.pone.0034561

**Published:** 2012-04-12

**Authors:** Mahesh Mohan, Deepak Kaushal, Pyone P. Aye, Xavier Alvarez, Ronald S. Veazey, Andrew A. Lackner

**Affiliations:** 1 Division of Comparative Pathology, Tulane National Primate Research Center, Covington, Louisiana, United States of America; 2 Division of Bacteriology and Parasitology, Tulane National Primate Research Center, Covington, Louisiana, United States of America; Ulm University, Germany

## Abstract

**Background:**

The Gastrointestinal (GI) tract is critical to AIDS pathogenesis as it is the primary site for viral transmission and a major site of viral replication and CD4^+^ T cell destruction. Consequently GI disease, a major complication of HIV/SIV infection can facilitate translocation of lumenal bacterial products causing localized/systemic immune activation leading to AIDS progression.

**Methodology/Principal Findings:**

To better understand the molecular mechanisms underlying GI disease we analyzed global gene expression profiles sequentially in the intestine of the same animals prior to and at 21 and 90d post SIV infection (PI). More importantly we maximized information gathering by examining distinct mucosal components (intraepithelial lymphocytes, lamina propria leukocytes [LPL], epithelium and fibrovascular stroma) separately. The use of sequential intestinal resections combined with focused examination of distinct mucosal compartments represents novel approaches not previously attempted. Here we report data pertaining to the LPL. A significant increase (±1.7-fold) in immune defense/inflammation, cell adhesion/migration, cell signaling, transcription and cell division/differentiation genes were observed at 21 and 90d PI. Genes associated with the JAK-STAT pathway (IL21, IL12R, STAT5A, IL10, SOCS1) and T-cell activation (NFATc1, CDK6, Gelsolin, Moesin) were notably upregulated at 21d PI. Markedly downregulated genes at 21d PI included IL17D/IL27 and IL28B/IFNγ3 (anti-HIV/viral), activation induced cytidine deaminase (B-cell function) and approximately 57 genes regulating oxidative phosphorylation, a critical metabolic shift associated with T-cell activation. The 90d transcriptome revealed further augmentation of inflammation (CXCL11, chitinase-1, JNK3), immune activation (CD38, semaphorin7A, CD109), B-cell dysfunction (CD70), intestinal microbial translocation (Lipopolysaccharide binding protein) and mitochondrial antiviral signaling (NLRX1) genes. Reduced expression of CD28, CD4, CD86, CD93, NFATc1 (T-cells), TLR8, IL8, CCL18, DECTIN1 (macrophages), HLA-DOA and GPR183 (B-cells) at 90d PI suggests further deterioration of overall immune function.

**Conclusions/Significance:**

The reported transcriptional signatures provide significant new details on the molecular pathology of HIV/SIV induced GI disease and provide new opportunity for future investigation.

## Introduction

Human immunodeficiency virus (HIV) and simian immunodeficiency virus (SIV) infections are characterized by continuous CD4^+^ T cell destruction, chronic immune activation and increased susceptibility to opportunistic infections that are easily controlled by healthy individuals [1]. The gastrointestinal immune system, in particular, is an important target of HIV/SIV as it is not only the largest immunologic organ but also a major site for viral replication and CD4^+^ T cell destruction (as early as 21 days post infection) [2]–[6]. The loss of CD4^+^ T cells from the GI immune system is often associated with significant pathological alterations in GI structure and function [7]–[9]. The GI pathology, characterized by chronic persistent inflammation and a variety of histopathological abnormalities [7]–[8], is believed to set the stage for pathological events that lead to AIDS progression [10]. More specifically, breakdown of the intestinal epithelial cell barrier, a common occurrence in intestinal disease, was shown to facilitate translocation of intestinal lumenal bacteria and their products into the systemic circulation leading to chronic activation of the immune system and progression to AIDS [10]. While the exact chronological events that lead to intestinal epithelial barrier disruption remain to be determined, it is reasonable to assume that inflammatory cell infiltration in the lamina propria [7] and subsequent proinflammatory cytokine production [11] in response to viral replication can indirectly affect epithelial cell function including alterations in epithelial cell permeability.

Based on our earlier studies, the occurrence of GI disease in SIV-infected rhesus macaques is associated with constitutive activation of the JAK-STAT pathway (Janus Kinase-Signal Transducer and Activator of Transcription). More specifically, GI disease in SIV-infected rhesus macaques was accompanied by increases in IL-6 mRNA, constitutive activation of p-STAT3 and increases in SOCS-3 mRNA [12]. Expression of p-STAT3 was localized to CD68 expressing macrophages and scattered CD3^+^ lymphocytes in the GI tract of SIV-infected rhesus macaques with chronic diarrhea [12]. In a follow up study, we also found significant increases in the expression of C/EBPβ, a proinflammatory transcription factor, in the GI tract of SIV-infected macaques [13]. In addition to being proinflammatory, C/EBPβ has been shown to enhance viral replication. More strikingly, we observed GI inflammation and disease in 70% (7/10) of macaques that did not have any opportunistic infections suggesting that the effects could be attributable to SIV. Further the data also indicated an association between persistent GI inflammation and increased mucosal viral loads which was reflected by increased binding of C/EBPβ and p65 to the SIV LTR (long terminal repeat) in lamina propria leukocytes (LPLs) isolated from the colon [13]. In addition to our studies, molecular pathological changes in GI function in response to HIV/SIV during acute and chronic infection have been also been described by others in detail [14]–[18].

While dissecting individual pro-inflammatory signal transduction mechanisms can provide detailed insight into the molecular pathology, the very nature of these studies can make the entire process less efficient and time consuming. An alternative and a more efficient approach is to obtain expression profiles either at the mRNA or protein level on a genome wide scale and then focus subsequent research efforts on key molecules/signaling pathways of interest. In more recent years, the application of functional genomics approaches to GI tract biopsies from HIV-infected individuals and SIV-infected rhesus macaques has revealed significant reduction in the expression of genes regulating cell cycle, lipid metabolism, epithelial cell barrier and digestive functions [14]–[15]. Although these studies contributed significant molecular insights into AIDS pathogenesis we strongly believe that the quantity and quality of information obtainable from any high throughput approach can be profoundly increased and further expanded if complexity of tissue samples can be addressed. The intestinal wall can be divided into several functional and anatomic components with the key events in AIDS pathogenesis occurring in the mucosa. The mucosa can be further subdivided into 1) intestinal epithelial cells, 2) intraepithelial leukocytes (IELs), 3) lamina propria leukocytes (LPLs) and 4) fibrovascular stroma. Both SIV and HIV infection are known to cause massive loss of intestinal CD4^+^ T cells that reside primarily in the lamina propria [2]–[6]. This marked population shift causes marked changes in gene expression, which would obscure changes in other components of the intestine. In order to make the starting material less complex and maximize specificity and sensitivity of information gathering we have separated the different cellular/structural components of the intestine (epithelium, IELs, LPLs, fibrovascular stroma., etc) and analyzed gene expression in each component separately. From the pathogenesis perspective, this novel approach creates a unique opportunity to investigate and better understand the intricate relationship between viral replication, the host immune system and damage to the structural and functional components of the intestine. To accomplish this we obtained serial intestinal resections prior to infection and at 21 and 90d PI from the same set of animals. This allowed each animal to be its own control further enhancing the quality of the data. The time points chosen were selected as they represent the nadir of CD4^+^ T cell depletion (peak viremia is at 14 d PI but peak mucosal CD4^+^ T cell loss is a week later) and establishment of viral set point. Using this novel combinatorial approach we have identified unique transcriptional signatures related to key pathogenic events such as immune activation, inflammation, epithelial barrier disruption (intestinal microbial translocation), immune cell dysfunction and mitochondrial anti-viral signaling in intestinal LPLs at 21 (acute) and 90 (chronic) d PI.

## Results

### Viral load and depletion of mucosal CD4+ T cells

**Figure 1:**
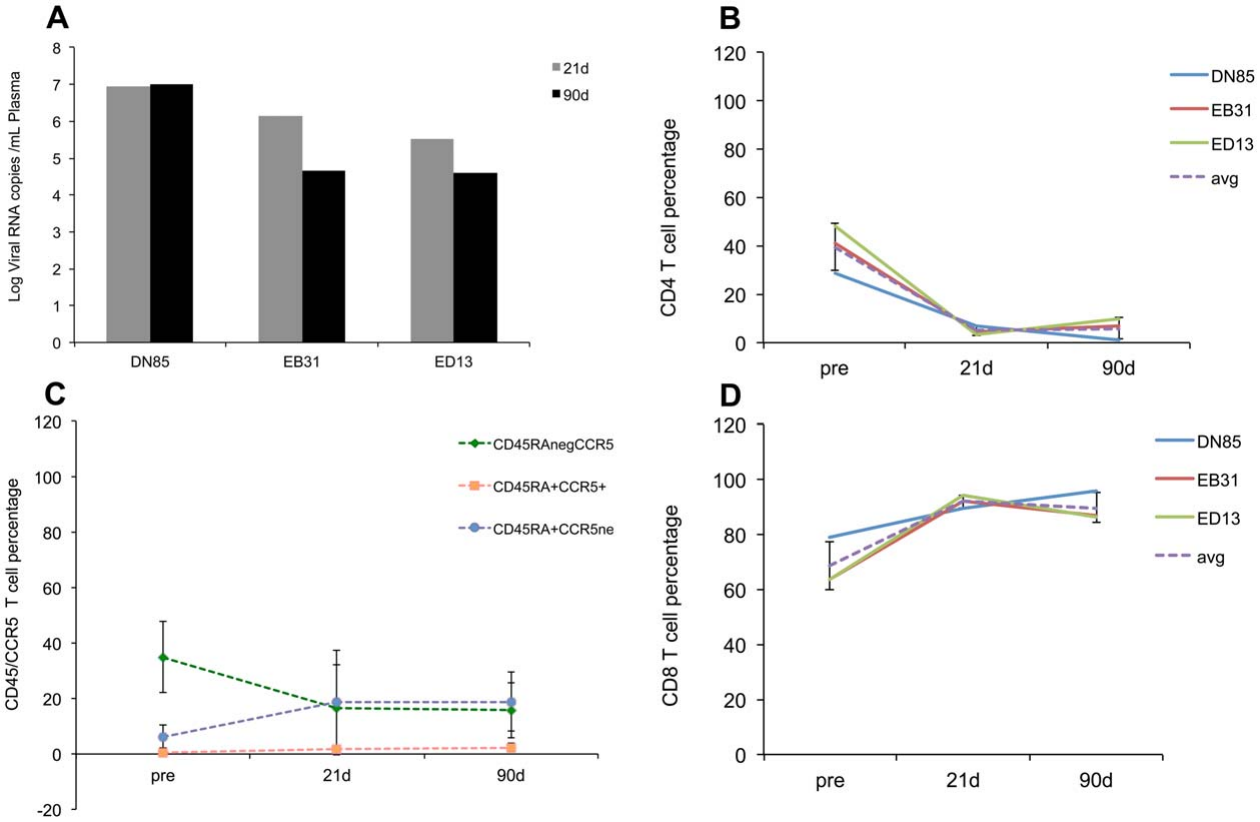
SIV infection results in elevated plasma viremia and rapid depletion of mucosal CD4+ T cells. Plasma viral loads (A) and changes in mucosal CD4+ T cells (B) “memory” CD4^+^ T cells (CD45RAneg, CCR5+) (C), and mucosal CD8^+^ T cells (D) in three Indian origin rhesus macaques at 21 and 90 days after intravenous infection with SIVmac251. Changes in CD45RA+/CCR5− populations at the 21 and 90d timepoints were not statistically significant (p>0.05).

Infection of rhesus macaques with SIV results in high acute plasma viral loads and rapid depletion of mucosal CD4^+^ T cells [2]–[6]. Consistent with these prior observations the animals used in this study had high viral loads (Fig. 1A) and a rapid and profound depletion of intestinal CD4^+^ T cells with a nadir at 21d after infection (Fig. 1B). Figure 1C further shows that the loss of CD4^+^ T cells was primarily due to depletion of the “memory” population that is CD45RA negative and CCR5 positive. The loss of mucosal CD4^+^ T cells was accompanied by a concomitant increase in CD8^+^ T cells (Fig. 1D) at 21 and 90d PI. Figure S1 shows total B cell percentages prior to and at 21 and 90d after infection. The average CD20^+^ B cell percentages (dotted line) at 21 and 90d after infection were not statistically different from the pre-infection time point.

### Gene expression profiles during acute SIV infection in the LPLs are indicative of early signaling events associated with immune cell differentiation, activation, expansion, migration and inflammation

Comparisons in gene expression in the LPLs [enriched for T and B lymphocytes (70–80%) but with substantial numbers of macrophages, NK, dendritic and plasma cells] obtained from the same animals prior to infection and at 21 and 90 days after infection were performed. Only those transcripts whose expression either increased or decreased by, at least, 1.7- fold (*P≤0.05*) in response to SIV infection were selected and considered differentially expressed.

Following analysis using DAVID [19]–[20] and GeneCards [21] we found 409 distinct transcripts to be upregulated in LPLs at 21d PI (Table 1). In contrast, the number of transcripts whose expression decreased at this time point was more than two fold greater (n = 858). At the 90 d PI, the number of transcripts with decreased expression (n = 1122) was nearly 3 times greater than the upregulated transcripts (n = 434). The fold difference and *p* values for a select number of differentially expressed (up and down) transcripts at 21 and 90 d PI are shown in tables 2 and 3. The entire list of differentially expressed genes in acute (21d) and chronic (90d) infection with their affymetrix IDs, p values and fold difference can be found in Table S1 and Table S2.

**Table 1:** Differentially expressed genes in lamina propria leukocytes (LPLs) during acute (21d PI) and chronic (90d PI) SIV infection.

	Up regulated	Known	Unknown	Annotated genes*
21 D PI	409	363	46	363
90 D PI	434	355	79	351

	Down regulated	Known	Unknown	Annotated genes
21 D PI	858	795	63	752
90 D PI	1122	938	134	910

* The numbers under the columns titled “Known” and “Annotated genes” differ as some of the annotated genes are represented by multiple probes.

**Table 2:** Select list of differentially expressed genes in LPL at 21 days post SIV infection.

Gene ID	Symbol	Fold Difference	P value
**Up vs Preinfection**			
Nuclear factor of activated T-cells, cytoplasmic, calcineurin-dependent 1	*NFATc1*	2.0	0.003
Signal Transducer and activator of Transcription 5A	*STAT5A*	2.8	0.008
E74-like factor 3 (ETS domain transcription factor)	*ELF3*	1.8	0.049
TAF5-like RNA polymerase II/P300/CBP-associated factor	*TAF5L/PCAF*	2.2	0.03
Aryl hydrocarbon receptor nuclear translocator	*AHRNT*	2.2	0.04
Estrogen receptor beta	*ESR2*	2.2	0.04
Myocardin	*MYOCD*	1.8	0.02
Nuclear receptor subfamily 6 group A	*NR6A1*	1.9	0.02
v-maf masculoaponeurotic fibrosarcoma oncogene	*MAF*	2.3	0.03
C-type lectin domain family 7, member A	*CLEC7A*	2.3	0.03
Toll-like receptor adaptor molecule	*TICAM2*	3.3	0.03
Complement component 1, s subcomponenet	*C1S*	5.2	0.04
Inhibitor of kappa light polypeptide gene enhancer in B cells epsilon	*IκBKE*	2.9	0.0008
Interleukin 10	*IL10*	2.4	0.02
Interleukin 12 receptor, beta 2	*IL12Rβ2*	2.8	0.03
Interleukin 21	*IL21*	1.9	0.04
Suppressor of cytokine signaling 1	*SOCS1*	1.9	0.02
Basement membrane-induced gene	*ICB1/C1orf38*	2.4	0.01
FYN binding protein	*FYB*	2.2	0.028
Arachidonate 5-lipoxygenase	*ALOX5*	4.0	0.002
CD6 antigen	*CD6*	3.2	0.02
Cyclin Dependant kinase 6	*CDK6*	2.4	0.04
Platelet derived growth factor beta polypeptide	*PDGFβ*	2.5	0.006
Oncostatin M precursor	*OSM*	2.8	0.01
Cardiotrophin-like factor 1	*CTLF-1*	3.8	0.01
Bone morphogenic protein 6	*BMP6*	2.6	0.02
Transforming growth factor beta 2	*TGFβ2*	2.0	0.03
Transforming growth factor beta 3	*TGFβ3*	2.4	0.02
Epidermal growth factor receptor	*EGFR*	1.8	0.02
Fibroblast growth factor 11	*FGF11*	3.0	0.04
Tumor necrosis factor receptor associated factor 4 isoform 1	*TRAF4*	2.5	0.02
v-akt murine thymoma viral oncogene homolog 2	*AKT2*	1.8	0.02
Catenin	*CTNND1*	2.1	0.008
Dystroglycan 1	*DAG1*	2.6	0.02
Integrin alpha L	*ITGAL*	1.8	0.02
Platelet/endothelial cell adhesion molecule (CD31)	*PECAM-1*	2.3	0.01
Mucin 5B	*MUC5B*	2.8	0.02
Gelsolin	*GSN*	5.7	0.02
Moesin	*MSN*	1.7	0.004
TBC1 domain family member 10A	*TBC1D10A*	1.8	0.03
**Down vs Preinfection**			
Interleukin 17D or Interleukin 27	*Il17D*	1.8	0.04
Interleukin 28B	*IL28B*	2.0	0.04
CCAAT-enhancer binding protein alpha	*C/EBPα*	2.2	0.02
Activation induced cytidine deaminase	*AID*	2.0	0.01
Peroxiredoxin 2	*PRDX2/NKEFB*	3.2	0.03
Granzyme K	*GZMK*	2.0	0.02
Interleukin 17 receptor E isoform 1	*IL17RE*	2.0	0.004
programmed cell death 2	PDCD2	2.0	0.04
programmed cell death 5	PDCD5	1.7	0.03
**Up vs 90 d PI**			
CD93	*CD93*	2.9	0.003
Cell adhesion molecule 1	*CADM1*	2.0	0.02
Intercellular adhesion molecule 1	*ICAM1*	1.7	0.02
Lectin galactose-binding soluble 3	*LGALS3*	2.0	0.02
CD40 ligand	*CD40LG*	2.8	0.03
Chemokine (C-C motif) ligand 3	*CCL3*	4.5	0.049
Chemokine (C-X-C motif) ligand 1	*CXCL1*	4.3	0.00
Interleukin 18	*IL18*	5.7	0.03
Tumor necrosis factor receptor superfamily, member 10b	*TNFRSF10B*	2.2	0.045
Tumor necrosis factor, alpha-induced protein 2	*TNFAIP2*	5.0	0.04
HLA class II histocompatibilty antigen, DM beta chain precursor	*HLA-DMB*	3.7	0.047
CD68	*CD68*	3.4	0.04
Suppressor of cytokine signaling 6	*SOCS6*	1.8	0.048

**Table 3:** Select list of differentially expressed genes in LPL at 90 days post SIV infection.

Gene ID	Symbol	Fold Difference	P value
**Up vs Preinfection**			
BCL6 co-repressor	*BCORL1*	2.5	0.04
SMAD6	*SMAD6*	2.8	0.04
Hepatocyte nuclear factor 4 alpha	*HNF-4α*	2.6	0.03
Chromodomain helicase DNA binding protein 1	*CHD1*	2.0	0.01
EPH receptor B1	*EPHB1*	2.1	0.01
Rho GTPase activating protein 1	*ARHGAP1*	3.3	0.02
Rho/Rac guanine nucleotide exchange factor 2	*ARHGEF2*	3.9	0.0007
Fibroblast growth factor receptor 1	*FGFR1*	3.2	0.001
c-jun N terminal kinase 3	*JNK3*	1.9	0.014
Suppressor of cytokine signaling 1	*SOCS1*	2.4	0.02
Fc fragment of IgA, receptor for	*FCAR*	1.9	0.03
Microtubule associated serine/threonine kinase 2	*MAST2*	4.4	0.04
Interleukin 1 receptor-like 1	*IL1RL1*	2.3	0.01
NLR family, pyrin domain containing 4	*NLRP4*	2.2	0.02
Nuclear factor of activated T cells 4	*NFAT4*	3.0	0.02
Tumor necrosis factor ligand superfamily member 7 (CD27 ligand) (CD70 antigen)	*CD70*	6.6	0.017
chemokine (C-X-C motif) ligand 11	*CXCL11*	2.4	0.003
Chitinase 1	*CHIT1*	4.0	0.047
CD109	*CD109*	1.8	0.026
Semaphorin 7A	*SEMA7A*	3.4	0.0006
Complement factor H	*CFH*	1.8	0.03
Lipopolysaccharide binding protein	*LBP*	5.3	0.02
Defensin beta 119	*DEFB119*	2.8	0.045
Tissue inhibitor of metallopeptidase 3	*TIMP3*	2.6	0.046
NLR family member X1	*NLRX1*	5.5	0.039
Fas-activated serine/threonine kinase	*FASTK*	2.9	0.02
**Down vs Preinfection**			
CD28 molecule	*CD28*	3.9	0.039
CD4 molecule	*CD4*	3.3	0.048
CD59 molecule, complement regulatory protein	*CD59*	2.6	0.005
CD86 molecule	*CD86*	4.5	0.03
CD93 molecule	*CD93*	3.1	0.04
Interleukin 1 receptor	*IL1R2*	3.0	0.003
T cell receptor beta chain V region CTL-L17	TRB@	2.0	0.005
Nuclear factor of activated T-cells, cytoplasmic, calcineurin-dependent 1	*NFATc1*	2.2	0.04
Interleukin 1 receptor associated kinase 3	*IRAK3*	2.6	0.02
Toll like receptor 8	*TLR8*	1.9	0.02
Toll like receptor adaptor molecule 2	*TICAM2*	2.5	0.03
Major histocompatibility complex, class II, DO alpha	*HLA-DOA*	5.5	0.03
Jun dimerization protein p21SNFT	*BATF3*	3.3	0.03
G protein-coupled receptor 183	*GPR183*	2.9	0.02
HLA class II histocompatibility antigen, DM beta chain	*HLA-DMB*	3.4	0.02
WD repeat domain 36	WDR36	3.7	0.01
**Up vs 21 d PI**			
Alpha defensin 2	*DEFA1*	2.5	0.019
Coagulation factor II (thrombin) receptor	*F2R*	2.1	0.005
Complement component 8 beta polypeptide	*C8B*	1.9	0.04
Cannabinoid receptor 2	*CNR2*	2.0	0.039
Thrombospondin 1	*THBS1*	1.8	0.038
CD38	*CD38*	1.8	0.015

**Figure 2:**
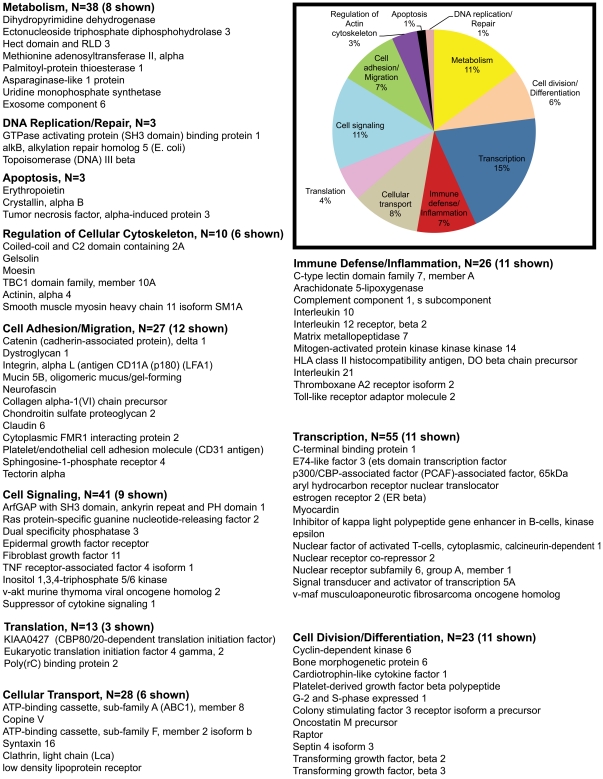
Gene functional categories up (1.7 fold) in LPLs at 21d PI. The relative size of each sector in the pie chart is determined by the number of genes in that functional category. Genes with unknown function are not included in the pie chart. Only a few transcripts of importance to SIV infection are shown in the figure under each functional category. The full list of genes grouped under each functional category for the 21d time point is provided in Table S1.

**Figure 3:**
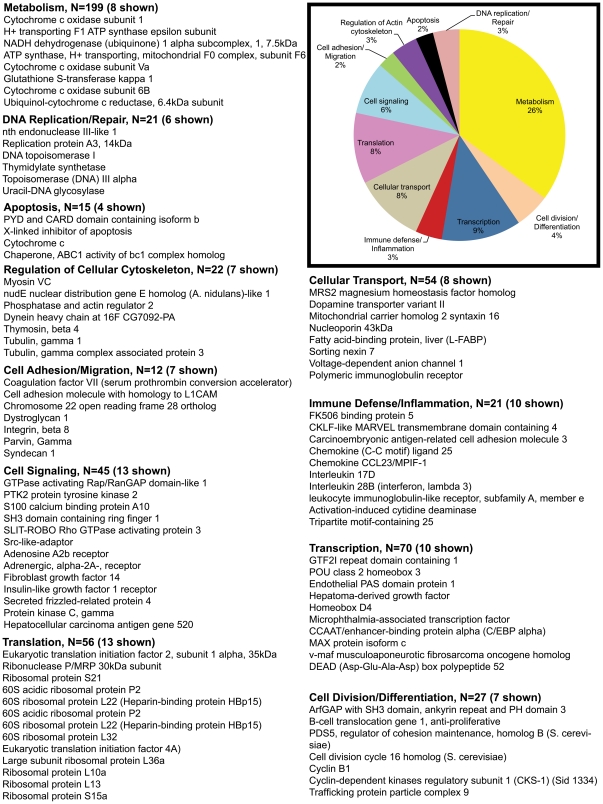
Gene functional categories down (1.7 fold) in LPLs at 21d PI. The relative size of each sector in the pie chart is determined by the number of genes in that functional category. Genes with unknown function are not included in the pie chart. Only a few transcripts of importance to SIV infection are shown in the figure under each functional category. The full list of genes grouped under each functional category for the 21d time point is provided in Table S1.

**Figure 4:**
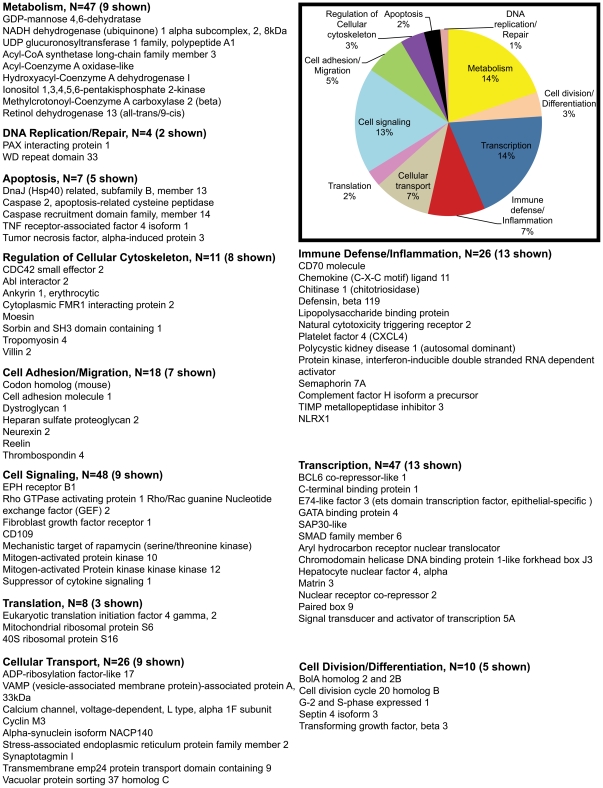
Gene functional categories up ((1.7 fold) in LPLs at 90d PI. The relative size of each sector in the pie chart is determined by the number of genes in that functional category. Genes with unknown function are not included in the pie chart. Only a few transcripts of importance to SIV infection are shown in the figure under each functional category. The full list of genes grouped under each functional category for the 90d time point is provided in Table S2.

**Figure 5:**
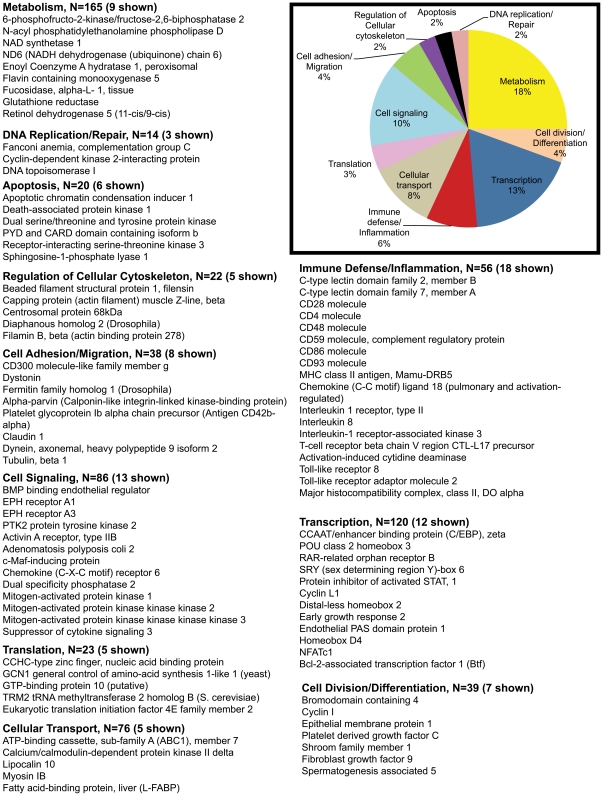
Gene functional categories down (1.7 fold) in LPLs at 90d PI. The relative size of each sector in the pie chart is determined by the number of genes in that functional category. Genes with unknown function are not included in the pie chart. Only a few transcripts of importance to SIV infection are shown in the figure under each functional category. The full list of genes grouped under each functional category for the 90d time point is provided in Table S2.

Out of 409 upregulated genes at 21d PI, DAVID identified 363 transcripts to be annotated (Table 1). Using both tools we broadly classified all differentially expressed genes into 10 different categories, namely; transcription, immune defense/inflammation, cell division/differentiation, cell signaling, cell adhesion/migration, transport, DNA replication/repair, regulation of cellular cytoskeleton, apoptosis, metabolism and transcripts with unknown function. The unknown transcripts are not represented in the pie charts shown in figures 2, 3, 4, 5.

Based on gene ontology/annotation, the 21d time point (the nadir of CD4^+^ T cell loss) yielded several interesting sets of altered genes. Genes associated with cellular transcription accounted for ∼15% (n = 55) of differentially expressed genes (Fig. 2). Of particular interest were *NFATc1 (T cell activation), STAT5A (IL-2, IL-15, IL-21 signaling), ETS domain transcription factor (macrophage activation), p300/CBP-associated factor (PCAF) associated factor (histone acetylase), aryl hydrocarbon receptor nuclear translocator (AHRNT) (immunosuppressant), estrogen receptor β (B cell activation), Myocardin, Nuclear receptor subfamily 6 (Co-repressor), group A, and v-maf (musculoaponeurotic fibrosarcoma oncogene) (coactivator)*.

Immune defense/inflammation and Cell division/differentiation accounted for ∼7% (n = 26) of the upregulated genes (Fig. 2). Prominent members in the immune defense/inflammation category included *C-type lectin domain family 7, member A (DECTIN1), Toll-like receptor adaptor molecule (TRIF/TRAM), Complement component 1, s subcomponent, inhibitor of kappa light polypeptide gene enhancer in B-cells epsilon (IκBKE, a corepressor), interleukin 10 (IL-10) (anti-inflammatory), interleukin 12 receptor, beta 2 (IL-12Rβ2) (Th1 response and proinflammatory) and interleukin 21 (IL-21) (proinflammatory)*. Notable genes regulating cell division/differentiation (6%, n = 23) included *CDK6, platelet derived growth factor beta polypeptide, oncostatin M precursor (OSM), cardiotrophin-like factor 1 (B cell activation), BMP6, TGF-β2 and TGF-β3 (cell differentiation)*.

Approximately, 11% of the transcripts included cell signaling genes (n = 22) such as *EGFR, FGF-11, TNF receptor associated factor 4 isoform 1, v-akt murine thymoma viral oncogene homolog 2, suppressor of cytokine signaling 1 (SOCS1 (Negative regulator of JAK-STAT pathway)*. Another 7% included genes associated with cell adhesion/migration (n = 27) that included *catenin, dystroglycan 1, integrin alpha L (leukocyte recruitment/trafficking), platelet/endothelial cell adhesion molecule (CD31 antigen) (leukocyte recruitment/trafficking), and mucin 5B*. About 3% (n = 10) of the transcripts represented genes regulating cellular cytoskeleton (*gelsolin, Moesin, TBC1 domain family member 10A, actinin, and alpha 4 smooth muscle myosin heavy chain 11 isoform SM1A*). The remaining transcripts fell into the following functional categories: metabolism (10%, n = 38), cellular transport (8%, n = 28), Apoptosis (1%, n =  3), DNA replication/repair (1%, n =  3), and translation (4%, n = 13) (Fig. 2).

In addition, compared to the 90d PI time point, the 21 day timepoint had increased expression of several genes associated with cell adhesion/migration [*CD93, cell adhesion molecule 1, intercellular adhesion molecule 1 (ICAM1), lectin galactoside-binding soluble 3*] and immune defense/inflammation [*CD4, CD40 ligand, Chemokine (C-C motif) ligand 3 (CCL3)*, Chemokine (C-X-C motif) ligand *1 (CXCL1), interleukin 18 (IL-18)*, *tumor necrosis factor receptor superfamily, member 10b (tnfrsf10b), tumor necrosis factor, alpha-induced protein 2, HLA class II histocompatibility antigen, DM beta chain precursor (MHC class II antigen DMB), CD68, ATP-dependant helicase LGP2, SOCS3, SOCS6*].

Collectively, the transcriptional profile presents clear evidence of increased transcription, inflammatory cytokine signaling, and metabolic changes associated with immune cell activation including increased leukocyte trafficking to sites of viral replication/inflammation. While immune cell activation is required to elicit a successful immune response against an invading pathogen, failure to moderate this response can cause significant injury to the host. Consequently, the increased transcription of genes with immunosuppressive function like *nuclear receptor co-repressor, IκBKE* (both transcriptional inhibitors), *IL-10* (an anti-inflammatory cytokine), *SOCS1* (negative regulator of JAK-STAT pathway) and *TGF-β1 & 2* serve to ensure that these responses are negatively regulated at multiple stages and that the host is protected from the adverse effects of an exuberant immune response.

### Genes encoding proteins linked to oxidative phosphorylation and antimicrobial/antiviral response are considerably downregulated in GALT during acute SIV infection

Interestingly, in contrast to the upregulated genes (n = 409) more than twice as many (n = 858) were downregulated at 21d PI (Table 1). 752 of these downregulated genes were annotated (Table 1). The exact reasons for this considerable decrease in gene expression are unclear but could be attributable to the massive virus induced CD4^+^ T cell loss that occurs by 21d PI (Fig. 1B). More importantly, a greater percentage of the downregulated transcripts represented genes regulating cellular metabolism (26%, n = 199). Within this category 29% (n = 57) were genes connected to oxidative phosphorylation/citric acid cycle. T cells switch from oxidative phosphorylation to glycolysis to meet their energy requirements in response to cellular activation [22]. These findings are very significant as it provides yet another novel transcriptional signature indicative of T cell activation as early as 21d PI. Another noteworthy gene downregulated at the 21 d time point was *peroxiredoxin* that may contribute to the antiviral activity of CD8^+^ T-cells [23].

Compared to the upregulated group, the percentage of genes regulating transcription (9%, n = 70), cell signaling (6%, n = 45), immune defense/inflammation (3%, n = 21), cell adhesion/migration (2%, n = 12), and cell division/differentiation (4%, n = 27) dropped significantly in the downregulated category (Fig. 2 & 3). On the other hand, the percentage of genes representing translation (8%, n = 56), apoptosis (2%, n = 15) and DNA replication/repair (3%, n = 21) showed a modest to substantial increase (Fig. 2 & 3). Percentage of genes regulating cellular transport and regulation of cellular cytoskeleton remained the same. Downregulated genes critical to HIV/SIV pathogenesis include *IL-17D, (also known as IL-27) and IL-28B (both anti-viral)*. *IL-17D* has sequence similarity to IL-17 and is produced by Th17 cells [24]. The massive depletion of Th17 cells during acute SIV infection might explain the reduced expression of its mRNA early in infection [25]. More importantly, *IL-17D or IL-27* inhibits HIV-1 replication in CD4^+^ T cells and macrophages [26]. Similarly, IL-28B in combination with IL-29 is deemed to be essential for mounting an efficient antiviral response [27]. Another notable gene that also displayed decreased expression is C/EBPα, previously shown to be downregulated by inflammatory cytokine signaling [28]. These observations suggest that in addition to immune cell activation, acute SIV infection is also characterized by a significant dampening of the antiviral response in the lamina propria.

### Transcriptional profile at viral set point infection is indicative of intestinal microbial translocation in conjunction with inflammatory signaling and progressive immune cell activation and dysfunction

At viral set point (90d PI) a total of 434 genes were found to be up-regulated (Table 1). Among these 351 were annotated genes (Table 1). Genes regulating transcription accounted for 14% (n = 47) of those upregulated (Fig. 4). Table 3 shows fold difference and *p* values for select transcripts important to HIV/SIV infection. Of particular interest were (*Bcl6 co-repressor (transcriptional repression), Aryl hydrocarbon receptor nuclear translocator (AHNT) (immunosuppressant), SMAD6 (anti-inflammatory), STAT5A (IL-2 signaling and T cell proliferation and clonal expansion), HNF-4α (macrophage activation) and Chromodomain helicase DNA binding protein 1 (transcriptional activator)*.

Cell signaling genes comprising *EPH receptor B1, Rho GTPase activating protein 1, Rho/Rac guanine nucleotide exchange factor 2, FGFR1, MAPK10 or JNK3 (c-jun N terminal kinase3 (pro-inflammatory), CD109 (negative regulation of TGFβ signaling), SOCS1 (negative regulator of JAK-STAT pathway)* comprised ∼13% (n = 48) of the upregulated genes (Fig. 4). Interestingly, several of the GTP binding proteins (*Rho GTPase activating protein 1, Rho/Rac guanine nucleotide exchange factor 2*) that play important roles in T cell activation are target genes induced by *STAT5A*, a downstream transcription factor activated by IL-2 signaling [29] which was also up-regulated during acute and chronic infection.

Approximately, 7% (n = 25) of the genes represented immune defense and inflammation. Notable genes were *CD38, CD70, CXCL11, chitinase1 (all 4 proinflammatory), semaphorin 7A, complement factor H, lipopolysaccharide binding protein (LBP) (proinflammatory), NLRX1 (negative regulator of mitochondrial anti-viral signaling), defensin beta (anti-microbial) and tissue inhibitor of metallopeptidase 3 (anti-inflammatory)* (Fig. 4). Semaphorin 7A (CD108) expressed on activated T cells has been shown to stimulate proinflammatory cytokine production in monocytes and macrophages via binding to α_1_β_1_ integrin (very late antigen-1) and is believed to be a mechanism by which effector T cells induce persistent inflammation through macrophage activation [30].

**Figure 6:**
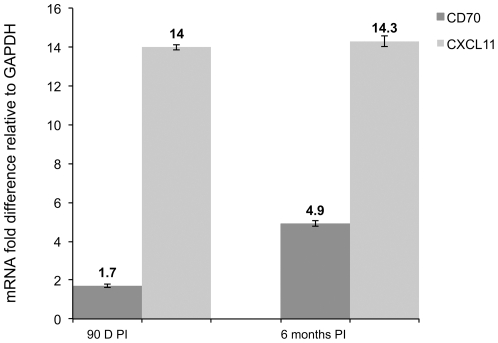
CD70 and CXCL11 expression is significantly increased in the LPLs in chronic SIV infection. Relative abundance in gene expression for CD70 (light bars) and CXCL11 (dark bars) in the lamina propria cellular compartment of the jejunum at 90 d (n = 3) and 6 months (n = 3) post SIV infection detected using quantitative real-time SYBR green two-step RT-PCR. The fold differences in gene expression were calculated as described in *Materials and Methods*. The relative fold increase is shown on top of each bar graph. The asterisk (*) indicates statistical significance (p<0.05).

We chose CD70 and CXCL11 for further confirmation studies using real-time RT-PCR because of their importance to HIV/SIV pathogenesis. CD70 has been previously linked to B cell dysfunction [31]–[32] and CXCL11 also known as interferon gamma inducible T cell alpha chemoattractant (ITAC) is a major chemokine shown to be produced by HIV infected macrophages for the recruitment of CCR5/CD4^+^ positive T cells as these cells also express CXCR3 (receptor for CXCL11) [33]. As shown in figure 6, quantitative real-time RT-PCR confirmed a statistically significant increase in the expression of CD70 (6 months post SIV infection) and CXCL11 (90d and at 6 months post SIV infection) in the lamina propria cellular compartment.

The remaining genes fell into the following categories: cell adhesion/migration (5%, n = 18), cellular transport (7%, n = 26), cell division/differentiation (3%, n = 10) regulation of cellular cytoskeleton (3%, n = 11), apoptosis (2%, n = 7), translation (2%, n = 8) and DNA replication/repair (1%, n = 4). Among the apoptotic genes *Fas-activated serine/threonine kinase* is striking, as it is a strong inducer of lymphocyte apoptosis [34].

In comparison to the 21d time point, several interesting genes associated with immune defense/inflammation (*alpha defensin 2, coagulation factor II (thrombin) receptor, complement component 8 beta polypeptide, cannabinoid receptor 2 (macrophage)*, cell adhesion/migration (thrombospondin 1) and cell signaling *(CD38, also an immune activation marker)* showed enhanced expression at the 90d time point post SIV infection.

Two other upregulated genes, namely, SMAD6 [35] and SOCS1 [36] are well established negative regulators of TGFβ/BMP and JAK-STAT pathways, respectively. In addition, SMAD6 also serves an anti-inflammatory function by interacting with the adaptor protein pellino-1 [37]. This may be viewed as a protective response to offset the increased expression of genes associated with inflammation and immune activation. While negative regulation is necessary to prevent uncontrolled cell signaling, paradoxically, the latter response could prove counterproductive to the host as it dampens the host's immune response to continuing viral replication, especially, by inhibiting anti-viral signaling by IFNγ [36].

### Genes associated with T cell signaling and innate immune responses are downregulated suggesting, immune cell exhaustion and dysfunction by 90 days post SIV infection

The 90d time point witnessed the maximum number of downregulated genes (1122 genes). Most of the downregulated genes fell into three categories: Metabolism (18%, n = 165), transcription (13%, n = 120), and cell signaling (10%, n = 92). The rest of the genes fell into the following categories: cellular transport (8%, n = 76), immune defense/inflammation (6%, n = 56), cell division/differentiation (4%, n = 39), cell adhesion/migration (4%, n = 38), regulation of cellular cytoskeleton (2%, n = 22), translation (3%, n = 23), DNA replication/repair (2%, n = 14) and apoptosis (2%, n = 20) (Fig. 5).

Interestingly, a considerable number of downregulated genes were associated with T cell signaling and activation such as *CD28, CD4, CD59, CD86, CD93, T-cell receptor beta chain V region CTL-L17, NFATc1* and innate immune responses such as Toll-like receptor (TLR) signaling (*IRAK3, TLR8, Toll like receptor adaptor molecule 2*), *major histocompatibility complex-DO alpha*, *interleukin 1 receptor*, including the proinflammatory/anti-HIV neutrophil chemoattractant *IL-8*. Together with the decline in CD4^+^ T cell numbers, the downregulation of *TLR8*, *IL-8* and *HLA-DOA* expressed on macrophages/dendritic cells and B cells, respectively, suggests generalized immune dysfunction that can in due course lead to immune exhaustion.

## Discussion

The intestine is a complex organ with multiple functional and structural elements made up of diverse cell types that closely interact with each other to perform complex functions with the single main objective of maintaining homeostasis [38]. Intestinal homeostasis is drastically altered during HIV/SIV infection as a result of massive loss of mucosal CD4^+^ T cells which then leads to structural and functional damage [7]–[9]. This sets the stage for development of a vicious cycle involving increased epithelial permeability, microbial translocation, localized inflammation, and localized and systemic immune activation [10]. Even though several previous high throughput studies have attempted to elucidate the molecular mechanisms, successful elucidation of the initiating and perpetuating mechanisms can be challenging in light of the marked shifts in cellular populations (loss of CD4^+^ T cells) limited primarily to one compartment (lamina propria) of the intestine (Fig. 1B&C). Tissue or sample complexity has long been recognized as a key factor influencing the success of a microarray experiment [39]. Accordingly, in whole tissues, changes in expression of key genes can be diluted or neutralized by unequal contribution of transcripts from multiple tissue compartments or cell types [39]. Secondly, valuable information can also be lost or masked due to differential regulation of gene expression within individual cell populations representing distinct tissue compartments (eg lamina propria vs. epithelium) [39]. As the different mucosal compartments in the intestine have their own function and accordingly a different and independent gene expression profile we have focused on LPLs in this manuscript for the following reasons. First, the LPLs offer a key line of defense to invading pathogens and foreign antigens. Second, this compartment contains the largest population of target cells for HIV/SIV infection [1]–[2]. Third, this compartment actively participates in mediating uncontrolled inflammation [12]–[13] that can lead to epithelial barrier disruption, a hallmark of untreated HIV/SIV infection. Fourth, our previous studies on proinflammatory signaling identified both lymphocytes and macrophages to be the major cell types associated with dysregulated signaling of the JAK-STAT3-C/EBPβ pathway [12]–[13]. Using this novel approach, apart from the broad range of innate immune mechanisms common to persistent inflammatory conditions our findings provide significant new clues on the potential mechanisms underlying critical pathogenic events such as immune cell activation, B cell dysfunction, microbial translocation, target cell recruitment and attenuated mitochondrial antiviral signaling.

Acute HIV/SIV infection is accompanied by explosive viral replication and severe CD4^+^ T cell destruction in mucosal tissues, particularly, GI associated lymphoid tissue (GALT) [2]–[6]. Interestingly, the critical pathogenic events such as immune cell activation, differentiation, expansion, migration, and activation of innate defense responses as well as counterbalancing anti-inflammatory responses were clearly reflected in the detailed transcriptional profile we generated from the LPL compartment at 21d PI. Immune cell activation (T, B, macrophages and other cells) and subsequent proliferation is dependent on increased transcription of new genes mediated by the activation of specific transcription factors [40]–[41]. This is clearly evident from the significant upregulation of several transcription factors in LPL's during acute infection, namely, *NFATc1*, *STAT5A*, *ESE-1*, *AHRNT* and *ERβ*. Among these *NFATc1* [42] and *STAT5A* [43], are known to regulate early processes in T cell activation. Activation of *NFATc1* in response to CD3 and CD28 co-stimulation is required for T cells to execute their effector functions including transcription of the *IL-2* gene that controls most of the early lymphocyte proliferative responses [44]. Signaling via *STAT5A*, a downstream transcription factor activated by IL-2, IL-12, IL-15 and IL-21, is required for cell cycle progression and clonal expansion of T cells [43]. Similarly, *E74 like factor-3 or ESE-1*, an ETS domain transcription factor with, at least, 30 members is induced in cells of the monocyte-macrophage lineage in response to inflammatory cytokines and lipopolysaccharide [45]. Further, *ESE-1* has been shown to enhance the transcription of proinflammatory proteins such as nitric oxide synthase [46] and angiopoietin [47]. Finally, *ERβ* is predominantly expressed in B cells and its engagement promotes B cell activation and survival [48]. The increased expression of select transcription factors with established roles in immune function suggests widespread immune activation in response to a rapidly replicating virus and is consistent with prior studies [15]–[17].

Lately, the activation of aryl hydrocarbon receptor (*AHR*) and its heterodimeric partner aryl hydrocarbon receptor nuclear translocator (*AHRNT*) has been shown to impact anti-viral immune defenses [49]. AHR activated in response to environmental signals translocates to the nucleus as a receptor-ligand complex, dimerizes with *AHRNT* after which the heterodimer binds to xenobiotic response elements on the DNA and induces the transcription of genes such as CYP1A1 [49]. Preliminary evidence from studies using the mouse model of human influenza A revealed suppressed lymphocyte responses and increased inflammation in the affected lung in response to AHR activation. Unfortunately, there is no information available on the role of *AHR* and *AHRNT* in HIV/SIV infection. However, given that LPS treatment markedly increased *AHR* and *AHRNT* mRNA expression in murine B cells and splenocytes [50], it is possible that low level LPS translocation from a leaky intestinal epithelial barrier early in infection might provide the stimulus for AHRT gene activation. Even though transcription factors for the most part regulate transcription, this process is largely dependent on the availability of a transcriptionally permissive chromatin. The increased expression of *p300/CBP-associated factor (PCAF)*, a histone acetylase [51], and *v-maf*, a transcriptional co-activator [52] that recruits *PCAF* to the gene promoters satisfies this critical requirement so that enhanced gene expression during immune cell activation can be successfully accomplished. Lastly, in addition to transcription factors, increased expression of gelsolin (actin regulatory protein) [53] and moesin (link proteins to the actin cytoskeleton) [54], two proteins that play critical roles in regulating the actin cytoskeleton during T cell activation and polarization further points toward immune cell activation early in SIV infection.

Apart from *STAT5A*, at least, six other differentially expressed genes, namely, *IL-12Rβ2*, *IL-21*, *IL-10*, *SOCS-1*, *cardiotrophin-like cytokine factor 1* and *oncostatin M* coupled to the JAK-STAT pathway were significantly up-regulated in LPL's during acute infection. *IL-12Rβ2* is expressed on activated T cells and is the predominant receptor that transduces IL-12 signals to effect a Th1 type immune response by activating STAT4 [55]. Apart from *IL-12Rβ2*, another gene that showed enhanced expression was *IL-21*, a pro-inflammatory cytokine that belongs to the common γ-chain-dependant family of cytokines [56]–[57]. *IL-21* has been shown to regulate the differentiation and function of effector CD4^+^ T helper cells, promote B cell differentiation and immunoglobulin production, and stimulate NK cell and CD8^+^ T cell cytotoxic function [56]–[57]. Further, *IL-21* can inhibit inducible T regulatory cell (Tregs) differentiation and reduce the ability of CD4^+^ T cells to respond to Treg-induced immunosuppression [56]–[57]. Furthermore, *IL-21* is required for Th17 cell differentiation and stimulates the expression of matrix degrading metalloproteases by intestinal fibroblasts and epithelial cells [56]–[57]. Serum levels of *IL-21* are significantly reduced in HIV-infected individuals early in infection and positively correlated with CD4^+^ T cell counts [58]. Since the primary source of IL-21 has been reported to be CD4^+^ T cells, the increased expression of this cytokine at this time point is puzzling and at the same time interesting as intestinal CD4^+^ T cells and Th17 cells are massively depleted as early as 21 d PI [2], [25]. Nonetheless, NK cell numbers markedly increase during acute SIV infection [59] and have been reported to be an alternative source of IL-21 [60]. The enhanced expression of IL-21 *in vivo*, in the GALT early in infection might serve as a transient immune enhancing mechanism to restore the massively depleted CD4^+^ T and Th17 cell populations. Other JAK-STAT activating cytokines such as *cardiotrophin like factor 1* can activate B cells [61]. *Oncostatin M* has been demonstrated to suppress colitis in DSS treated mouse models [62]. SOCS proteins negatively regulate the JAK-STAT pathway and the increased expression of SOCS-1 ensures that signaling via the JAK-STAT pathway does not remain constitutively active [36]. From these observations it is clear that activation of the JAK-STAT pathway occurs early in infection and provides a direct mechanism to stimulate processes such as cell activation, proliferation and differentiation that are critical to an immune response.

The success of a rapid inflammatory response is to a great extent reliant on the host's capacity to recruit immune/inflammatory cells to the site of viral replication/insult. Cell adhesion molecules such as *integrin alpha L (CD11a)* and *PECAM 1 (CD31)* play important roles in leukocyte recruitment via interactions with endothelial cells [63]–[64]. The integrin alpha chain (CD11a) in combination with the beta chain forms the functional *CD11a* molecule which serves as a receptor for Intercellular adhesion molecule 1 and 2 (ICAM) expressed mostly on endothelial cells [63]–[64]. Similarly, *PECAM-1* expression on leukocytes facilitates their directional migration along a chemokine gradient to inflammatory sites [65]. Taken together, the simultaneous up-regulation of both *CD11a* and *CD31* would suggest the presence of an active inflammatory response, a process that contributes directly to immune activation.

Another hallmark of T cell activation is that, naïve T cells reprogram their metabolism by switching from oxidative phosphorylation (OXPHOS) to glycolysis to meet their increasing energy demands to carry out various biosynthetic processes [22]. Consistent with this finding, we observed the downregulation of ∼57 genes encoding proteins belonging to the OXPHOS pathway. Unlike C/EBPβ, C/EBPα expression is significantly downregulated by inflammatory cytokines including lipopolysaccharide [28]. In agreement with the above finding, the decreased expression of C/EBPα observed in the present study indirectly indicates activation of inflammatory cytokine signaling in the LPL during acute SIV infection. Further, we observed significant down-regulation of two novel molecules with antiviral properties, namely, *IL-17D* or *IL-27* and *IL-28B* [26]–[27]. *IL-17D* or *IL-27* exerts its anti-viral effect by inducing a gene expression profile similar to that of IFN-α which includes APOBEC3G, an endogenous anti-viral protein [26]. Similarly, *IL-28B* was recently shown to increase granzyme B loading and potentiate CTL killing function in macaques [66]. To our knowledge, these novel observations uncovered by minimizing tissue complexity have not been reported previously *in vivo* and provide more evidence for T cell activation and increased susceptibility to GI related opportunistic pathogens early in infection.

The day 90 time point yielded the maximum number of differentially expressed genes (n = 1556). Similar to the acute stage, expression of transcription factors, namely, *ESE-1*, *AHRNT* and *STAT5A* continued to remain elevated during the chronic stage. Another markedly up-regulated transcription factor, *HNF-4α*, has been previously shown to be expressed abundantly by peritoneal macrophages where it induced the expression of fibroleukin, a protein linked to the pathogenesis of hepatic failure [67]. The immune defense/proinflammatory genes, included the ubiquitous gene encoding for *lipopolysaccharide binding protein* (*LBP*), an acute response protein that is predominantly synthesized by the liver but also produced by other tissues including the intestine, possibly, by macrophages in response to the presence of LPS [68]. The finding is important as it not only provides indirect evidence of intestinal bacterial translocation but also adds credence to the microbial translocation theory proposed as a cause of chronic immune activation that drives progression to AIDS [10]. Interestingly, LPS also directly induces the expression of *ESE-1* [45], *AHRNT* [50] and *STAT5A* [69] which were also elevated. Apart from LBP, other notable proinflammatory genes showing enhanced expression were *CD38*, *CD70*, *CXCL11*, *chitinase1* and *natural cytotoxicity triggering receptor 2 (NCR2)*. *CD38* is a glycoprotein (ectoenzyme) expressed on activated T cells and also used as an activation marker for T cells [70]. Increased numbers of CD8^+^CD38^+^ T cells have been reported in chronic infection and coupled to disease progression in chronic HIV infection [71]–[72]. Interestingly, the activation marker density, number and proportion of CD8^+^CD38^+^ T cells has been shown to positively correlate with viral load in acute [70] and chronic HIV infection [73]. *CD70*, also known as *tumor necrosis factor (ligand) superfamily, member 7*, is expressed abundantly on activated T cells of HIV-infected patients [31]. It has been reported to contribute directly to hypergammaglobulinemia in HIV-infected patients by stimulating memory B cells via *CD27* and promoting their differentiation into plasma cells that subsequently produce elevated levels of immunoglobulin [31]. Overexpression of *CD70* in transgenic mice resulted in depletion of naïve T cell pools in the spleen and lymph nodes due to their continuous differentiation into effector T cells via *CD70*-*CD27* interactions [32]. These mice died of *penumocystis carinii* pneumonia, a hallmark of T cell immunodeficiency even in the absence of a lentiviral infection [32]. The chemokine, *CXCL11* is induced by interferons in a proinflammatory environment and plays a central role in recruiting CCR5/CD4^+^ T cells to HIV infected antigen presenting cells (macrophages and dendritic cells) and also their retention in lymph nodes of HIV-infected individuals [33]. The elevated expression of CXCL11 (∼14-fold) detected at 90 d and 6 months post SIV infection (figure 4) represents a robust host response that is effectively exploited by the virus to ensure constant recruitment of target cells to the intestinal lamina propria, a major site of viral replication.

Similarly, *chitinase1* expression is considerably increased in macrophages in inflamed tissues [74]. Also, *NCR2* is expressed on NK cells and activation of this receptor results in increased efficiency of NK cell function [75]. Additionally, *JNK3*, also known as *stress activated protein kinase* is a proinflammatory transcription factor activated by cytokines like TNF-α, IL-1β, growth factors and a variety of environmental stresses [76]. Its increased expression is well documented in colonic lamina propria cells of inflammatory bowel disease patients [77]. Lastly, the identification of *NLRX1*, a recently described regulator of mitochondrial antiviral immunity is yet another important new finding to emerge from this study. *NLRX1* was shown to inhibit anti-viral cytokine responses mediated through RIG-like helicase family of intracellular receptors and the mitochondrial anti-viral signaling (MAVS) adaptor [78]. siRNA induced knockdown of *NLRX1* promoted virus-induced type I interferon production and decreased viral replication [78]. Future studies are definitely required to further understand the role of *NLRX1* in HIV/SIV replication as it has a high potential to serve as a therapeutic target for decreasing viral replication. Collectively, the transcriptional signature at viral set point is suggestive of marked B cell dysfunction, increased expression of proinflammatory molecules, negative regulation of anti-viral signaling and widespread T cell activation, most likely, in response to translocation of intestinal bacteria and bacterial products.

Gene expression for several components of the T cell signaling pathway was considerably decreased during chronic infection. These included *CD28, CD4, CD59, CD86, CD93, interleukin 1 receptor, T-cell receptor beta chain V region CTL-L17, NFATc1, and c-type lectin domain family 2, member B (IFN-alpha-2b-inducing-related protein 1)*. Downregulation of *CD28* has been previously reported to occur in lymphoid tissues of HIV-infected patients who progressed to AIDS [79]. The decreased expression can be directly attributed to a combination of CD4^+^ T cell loss and decreased expression of genes in immune cells due to a dysfunctional immune response.

Interestingly, several immune mediators expressed exclusively by macrophages and B cells showed markedly reduced expression during chronic infection. These included the two pathogen recognition receptors *c-type lectin domain family 7*, *member A (DECTIN1)*, *TLR8*, their signaling components (*IRAK3, TLR8, Toll like receptor adaptor molecule 2*), *proinflammatory chemokines IL-8*, *CCL18*, *major histocompatibility complex class II*, *DO alpha* and *SOCS-3*. DECTIN1 and TLR8 are pattern recognition receptors that specifically recognize β-glucans [80] and single stranded RNA on bacteria/fungi and viruses, respectively [81]. Activation of *TLR8*, which is highly expressed on B cells, macrophages and immature dendritic cells, can suppress HIV-1 replication in lymphoid tissue of tonsillar origin [82]. Also, *IL-8*, a chemoattractant for neutrophils, basophils and T cells, was recently reported to inhibit HIV replication in PBMCs and ectocervical tissues [83]. While the mechanisms remain unclear, the downregulation of *IL17D* or *IL-27* (also downregulated in acute infection) might partially explain the reduced *IL-8* expression observed later in infection. Given the evidence that *IL-17D* can stimulate *IL-8* production [84], the continual destruction of Th17 cells throughout SIV infection [25] might abolish this stimulus leading to reduced *IL-8* production by GALT. Similarly, *CCL18*, a chemokine also synthesized by macrophages is a chemoattractant for naive T cells, CD4^+^ and CD8^+^ T cells and nonactivated lymphocytes [85]. Further, the reduced expression of *HLA-DOA*, a MHC class II molecule expressed on B cells suggests diminished immune responses (antigen presentation) during chronic infection [86]. Lastly, the downregulation of *SOCS3* a negative regulator of several cytokines that activate the JAK-STAT and TLR pathways suggests dysregulation resulting in constitutive activation of proinflammatory cytokine signaling [36]. On the whole, the reduced expression suggests continual T cell destruction without reconstitution, diminished innate immune mechanisms, and markedly dysregulated macrophage and B cell function, the latter well known to be major features of chronic HIV infection.

In summary, our findings provide significant new knowledge pertaining to the complex molecular events that unfold in the LPL compartment of the intestine during acute and chronic SIV infection. More importantly, minimizing tissue complexity enabled the successful *in vivo* identification of interesting transcriptional signatures associated with key pathogenic events occurring in the GALT separately at 21 and 90 d post SIV infection. These include significant new clues to the possible mechanisms underlying B cell dysfunction (*CD70, GPR183, AICDA*), T cell activation (*CD6, Semophorin 7A, reduced expression of genes linked to OXPHOS/Citric acid/TCA cycle*), macrophage dysfunction (*chitinase, DECTIN1, TLR8, CCL18, IL8*), antiviral signaling/activity (*IL17D, IL28B, NLRX1, CCL3, peroxiredoxin*), lymphocyte apoptosis (*FASTK*) and microbial translocation (*LBP, AHRNT, C/EBPα, ESE-1, STAT5A*). Interestingly, the increased expression of *ESE-1, STAT5A, AHRNT, LBP* coupled with decreased expression of *C/EBPα* indirectly suggests that microbial translocation is occurring early in infection even though data from peripheral blood show modest evidence of elevated LPS in circulation. Future studies involving flow cytometry, *in situ* hybridization and immunofluorescence are required to validate the specific cell types in the LPL compartment of the intestine that express these differentially expressed genes. This will significantly help streamline future research efforts on key molecules/signaling pathways of interest to HIV/SIV pathogenesis. Similar high throughput studies encompassing the intestinal epithelium, intraepithelial lymphocytes and fibrovascular stroma in the immediate future will add additional insight into the molecular mechanisms underlying GI dysfunction.

## Materials and Methods

### Ethics statement

All experiments using rhesus macaques were approved by the Tulane Institutional Animal Care and Use Committee (Protocol 3267-B00). The Tulane National Primate Research Center (TNPRC) is an Association for Assessment and Accreditation of Laboratory Animal Care International accredited facility (AAALAC #000594). The NIH Office of Laboratory Animal Welfare assurance number for the TNPRC is A3071-01. All clinical procedures, including administration of anesthesia and analgesics, were carried out under the direction of a laboratory animal veterinarian. Animals were anesthetized with ketamine hydrochloride for blood collection procedures. Intestinal resections were performed by laboratory animal veterinarians. Animals were pre-anesthetized with ketamine hydrochloride, acepromazine, and glycopyrolate, intubated and maintained on a mixture of isoflurane and oxygen. Buprenorphine was given intra-operatively and post-operatively for analgesia. All possible measures are taken to minimize discomfort of all the animals used in this study. Animals were closely monitored daily following surgery for any signs of illness such as anorexia, lethargy, diarrhea, vomiting, and dehydration. Appropriate medical care was implemented if any of these signs of illness were noted. Rhesus macaques can develop a fatal AIDS-like disease after infection with SIV. If euthanasia was required in the judgment of the TNPRC veterinary staff, animals were euthanized in accordance with the recommendations of the panel on Euthanasia of the American Veterinary Medical Association. Tulane University complies with NIH policy on animal welfare, the Animal Welfare Act, and all other applicable federal, state and local laws.

### Animals and Tissue Collection

Serial resection biopsies (∼6–8 cm long) of jejunum were collected from three Indian-origin rhesus macaques prior to infection and 21 and 90d after infection with SIVmac251 for microarray studies. For quantitative RT-PCR studies jejunal tissues from six additional SIV infected macaques and six uninfected control macaques were also examined.

### Cell isolation from Intestinal resection segments

In order to determine the impact of high viral replication and massive CD4^+^ T cell loss on the intestinal mucosa we conducted a longitudinal study to assess genome wide changes in gene expression profiles during SIV infection using Affymetrix (Santa Clara, CA) rhesus macaque arrays that contain about 54,675 capture probes. To minimize information loss and to make the starting material less complex we separated the intestinal epithelial cells from the underlying LPLs and fibrovascular stroma. Finally, the intra epithelial cells (IELs) were separated from the epithelial cells and changes in gene expression were analyzed in all 4 compartments separately. In order to successfully separate all 4 tissue compartments and ensure the availability of sufficient starting material we obtained intestinal resection segments (6–8 cm long) from the jejunum instead of pinch biopsies. To prioritize our efforts we have in the present communication focused on the changes occurring in the LPLs at 21 and 90 days after SIV infection. Comparisons in gene expression were made to resection segments collected from the same animal 6 weeks prior to SIV infection. While profiling gene expression in a single cell is the gold standard, performing such an analysis on the intestine can be a very painstaking process due to the fact that the intestine in its entirety comprises, at least, 20–25 different cell types. Accordingly, while the LPLs are enriched for lymphocytes (70–80%); this population also contains small numbers of macrophages, dendritic and plasma cells.

Briefly, surgical resection segments (6–8 cm long) for mRNA profiling studies were first incubated with vigorous shaking in Ca^++^Mg^++^ free-HBSS containing 1 mM EDTA for two 30-min incubations at 37°C to separate the intestinal epithelial cells [2], [87]. Following incubation, the epithelial cells in the supernatant were harvested by centrifugation at 500 g for 10 min followed by subjecting the cells to percoll density gradient centrifugation to separate IELs [2], [87]. After dislodging the epithelial cells the tissue segments were incubated twice for 30 minutes duration in RPMI1640 containing 20 U of collagenase per ml while rapidly shaking at 37°C to separate LPLs from the fibrovascular stroma [2], [87]. The tissue homogenates were subjected again to percoll density gradient centrifugation to separate LPLs from the fibrovascular stroma. The purified components were then used for flow cytometry and microarray hybridization as indicated below.

For quantitative RT-PCR confirmation studies described below, jejunal tissues from six SIV infected macaques (3 animals at 90d PI and 3 at 6 months PI) and six uninfected control macaques were processed similarly except that the tissue homogenates containing the lamina propria cellular population prepared after collagenase digestion were not subjected to percoll density gradient centrifugation and instead were used as such for total RNA extraction.

### Phenotyping blood and tissue mononuclear cells

Peripheral blood mononuclear cells (PBMCs) were isolated and processed as previously described [83]. PBMCs were collected by centrifugation over lymphocyte separation media. Cells (PBMCs and LPLs) were adjusted to a concentration of 10^7^/ml and 100 µl aliquots (10^6^ cells) were stained with appropriately diluted, directly-conjugated monoclonal antibodies to CD45RA fluorescein isothiocyanate (FITC), CCR5 and CD20 phycoeryrthrin (PE), CD8-peridinin chlorophyll A protein (PerCP) and CD4-allophycocyanin (APC) (all from BD Biosciences Pharmingen San Diego, CA). Samples were stained for 30 min in the dark at 4°C, fixed in 2% paraformaldehyde, and stored in the dark at 4°C overnight for acquisition the next day. Samples were acquired on a LSR II flow cytometry equipment (BD Biosciences) and analyzed with Flow Jo software (Treestar Inc, Ashland, OR). Samples were first gated on lymphocytes by forward and side scatter plots and then on CD4^+^ or CD8^+^ lymphocytes. Changes in CD45RA^+^/CCR5^−^ populations at the 21 and 90d timepoints were analyzed using the wilcoxon matched-pairs signed rank test.

### Microarray Hybridization and Statistical Analysis

Microarray-based profiling of genome wide changes in mRNA expression in LPL samples was performed using Affymetrix rhesus monkey GeneChips (U133A 2.0). RNA was isolated from the three LPL samples derived from intestinal resection seqments collected at 6 weeks before and at 21 and 90d post-SIV infection. Total RNA was used to synthesize double-stranded cDNA (Superscript Choice System; Life Technologies Bethesda Research Laboratories). The resulting cDNA was purified and used for *in vitro* transcription to produce biotin-labeled cRNA (BioArray HighYield RNA Transcription Labeling kit; Enzo Diagnostics). The biotinylated cRNA was cleaned (RNAeasy Mini kit; Qiagen), fragmented, and hybridized on GeneChips containing 54,675 probes sets, using standard protocols at a commercial GeneChip core facility (). Following three washes, individual GeneChips were stained with streptavidin-phycoerythrin (Molecular Probes), amplified using biotinylated anti-streptavidin (Vector Laboratories), and scanned for fluorescence (GeneArray Scanner; Hewlett Packard) measurement on a Microarray Suite 5.0 software (MAS 5.0; Affymetrix).

For data analysis, the Affymetrix CEL files (containing scanned images, together with absolute calls for each gene) were transferred to the S+ statistical module within the Spotfire DecisionSite for Microarray Analysis (TIBCO-Spotfire) program. Chips were normalized using the Robust Multichip Analysis (RMA) method, to stabilize MvA plots. This step was essential to eliminate any intensity-specific bias in probe-level data and to produce a matrix comprising of normally distributed data. Expression indices were reported as log (base 2) of change in gene-expression at either 21d or 90d time-points, relative to the 0 d baseline. Probe sets whose targets were not detected were removed from the data matrix. A Student's t test was then performed to identify genes expressed in a statistically significant manner (*P<0.05*). A fold change cutoff of ≥1.7-fold in all three NHPs was then applied, so as to only consider genes whose expression was perturbed in magnitude and in a statistically significant manner. The microarray data has been uploaded to Gene Expression Omnibus and the GEO accession numbers are GSM846893 through GSM846901.

Gene ontology/annotation analysis was performed using the DAVID (Database for Annotation, Visualization and Integrated Discovery) Bioinformatics Functional Annotation tool (http://david.abcc.ncifcrf.gov) [19]–[20] and GeneCards® (http://www.genecards.org/) [21] on all differentially (Up and Down) expressed transcripts.

### Quantitative Real-Time SYBR Green two-Step RT-PCR

Gene expression for CD70 and CXCL11 in the jejunal lamina propria cellular compartment of two SIV infected macaques was further evaluated by Quantitative Real-Time SYBR Green Two-Step RT-PCR assay (QRT-PCR) (ABI, Foster City, CA). Total RNA was extracted using the miRNeasy kit (Qiagen Inc, Valencia, CA) and reverse transcribed using the SuperScript. III First-Strand Synthesis System for RT-PCR kit following the manufacturer's protocol. Each QRT-PCR reaction (20 µl) contained the following: 2× Power SYBR Green Master Mix without uracil-N-glycosylase (12.5 µl), target forward and reverse primer (200 nM) and cDNA (4 µl). Forward and reverse primer sequence for CD70, CXCL11 and GAPDH is shown in Table 4. The PCR amplification was carried out in the ABI 7900 HT Fast PCR System (Applied Biosystems, Foster City, CA). Thermal cycling conditions were 95°C for 10 minutes followed by 40 repetitive cycles of 95°C for 15 sec, 60°C for 1 min. As a normalization control for RNA loading, parallel reactions in the same multiwell plate were performed using glyceraldehyde-3-phosphate dehydrogenase (GAPDH).

**Table 4:** Primer sequences used for real time Power SYBR Green Two-step RT-PCR.

Gene Name	Primer Sequence	Product size (bp)	Primer Concentration
CD70	For- 5′-TGACGGCATCTACATGGTCCACAT-3′Rev- 5′-GGTGGTGGTGTCTGGAGGTC-3′	77	200 nM
CXCL11	For-5′-ATGAGTGTGAAGGGCATGGCTA-3′Rev-5′-GAACATAGGGAAACCTTGAACAACCGTA-3′	75	200 nM
GAPDH	For-5′-TCCTGCACCACCAACTGCTTAG-3′Rev-5′-TGTGGTCATGAGTCCTTCCACGAT-3′	81	200 nM

Quantification of gene amplification following RT-PCR was made by determining the threshold cycle (C_T_) number for SYBR Green fluorescence within the geometric region of the semi-log plot generated during PCR. Within this region of the amplification curve, each difference of one cycle is equivalent to a doubling of the amplified product of the PCR. The relative quantification of target gene expression across treatments was evaluated using the comparative C_T_ method. The ΔC_T_ value was determined by subtracting the GAPDH C_T_ value for each sample from the target C_T_ value of that sample. Calculation of ΔΔC_T_ involved using the highest sample ΔC_T_ value (i.e., sample with the lowest target expression) as an arbitrary constant to subtract from all other ΔC_T_ sample values. Fold changes in the relative gene expression of target was determined by evaluating the expression, 2^−ΔΔ CT^. The data was analyzed using using RealTime StatMiner™ package, a bioinformatics software developed by integromics, on Spotfire DecisionSite.

## Supporting Information

Figure S1 **Percentages of B cells in the intestinal lamina propria prior to and at 21 and 90d after SIV infection.** The average CD20^+^ B cell percentages (dotted line) at 21 and 90d after infection were not statistically different from the pre-infection time point (p>0.05).
(TIF)

Table S1 **The full list of differentially expressed genes showing statistical significance at 21 days after SIV infection with their affymetrix IDs, gene and functional annotation.** Tab 1: Up in 21d PI, Tab 2: Down in 21d PI, Tab 3: Up in 21d compared to 90d PI.
(XLSX)

Table S2 **The full list of differentially expressed genes showing statistical significance at 90 days after infection with their affymetrix IDs, gene and functional annotation.** Tab 1: Up in 90d PI, Tab 2: Down in 90d PI, Tab 3: Up in 90d compared to 21d PI.
(XLSX)
