# Supplementary figures and images for: Focused Examination of the Intestinal lamina Propria Yields Greater Molecular Insight into Mechanisms Underlying SIV Induced Immune Dysfunction
Source: PLoS One. 2012 Apr 12;7(4):e34561. doi: 10.1371/journal.pone.0034561 (PMC3325268; doi:10.1371/journal.pone.0034561)

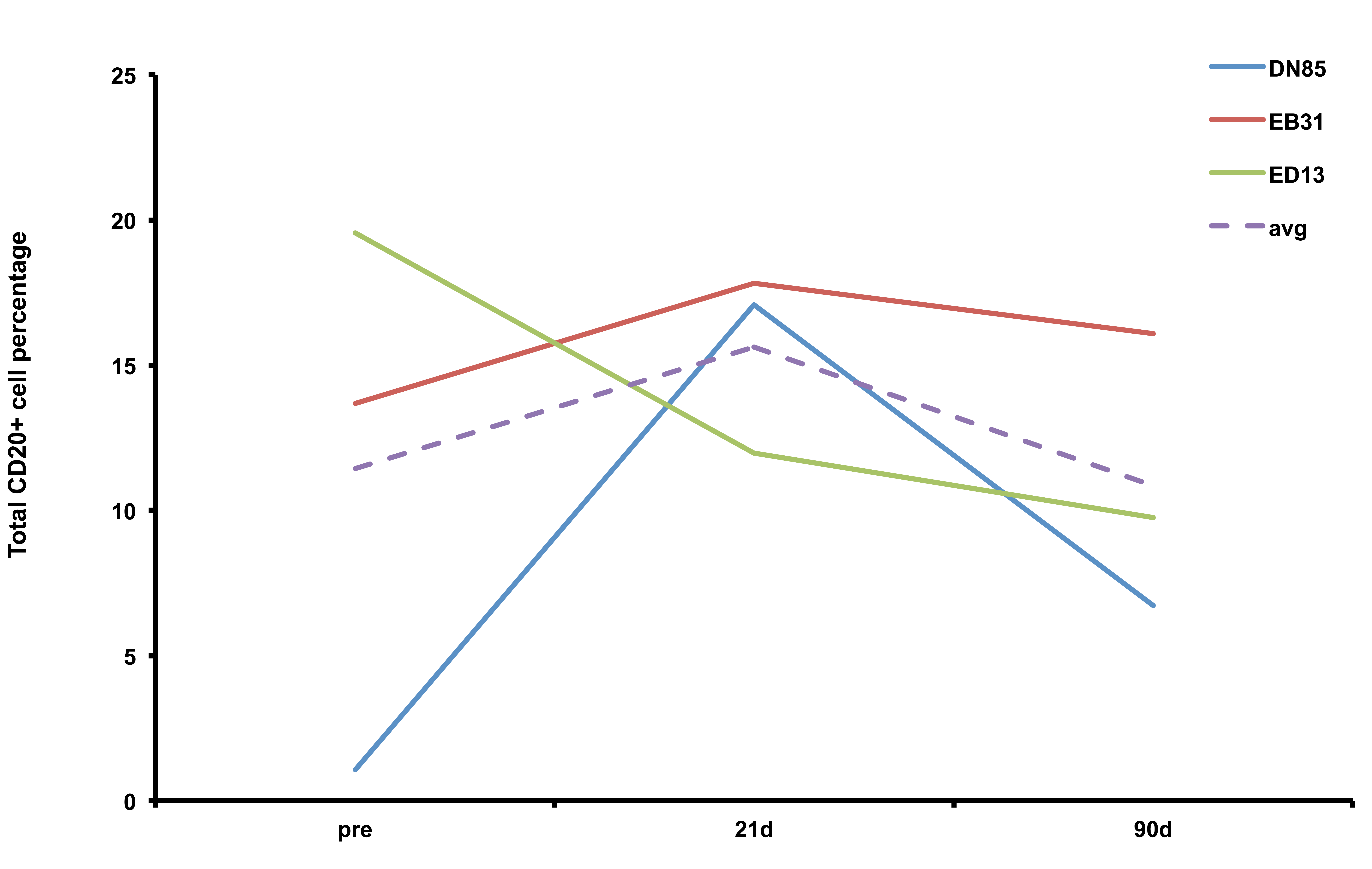

Supplement: Figure S1 — Percentages of B cells in the intestinal lamina propria prior to and at 21 and 90d after SIV infection. The average CD20+ B cell percentages (dotted line) at 21 and 90d after infection were not statistically different from the pre-infection time point (p>0.05). (TIF) [file pone.0034561.s001.tif]
